# Does life story work improve psychosocial well-being for older adults in the community? A quasi-experimental study

**DOI:** 10.1186/s12877-018-0797-0

**Published:** 2018-05-16

**Authors:** Claudia K. Y. Lai, Ayumi Igarashi, Clare T. K. Yu, Kenny C. W. Chin

**Affiliations:** 1 School of Nursing, The Hong Kong Polytechnic University, Yuk Choi Road, Hung Hom, Hong Kong SAR; 2 School of Health Sciences and Nursing, Graduate School of Medicine, The University of Tokyo, 7-3-1 Hongo, Bunkyo-ku, Tokyo, Japan; 3 Stat Solutions Co, Block A, 1/F., Wing Hin Factory Building, 31-33 Ng Fong Street, Sanpokong, Hong Kong

**Keywords:** Life story, Aged, Older adults, Community-dwelling, Mental health, Life satisfaction, Self-concept, Depression

## Abstract

**Background:**

Previous studies have demonstrated that life story work has positive effects when used on older adults. This study aimed to examine the effect of life story work on the general mental well-being, self-esteem, and life satisfaction of older adults by comparing two groups – one with and one without depressive symptoms.

**Methods:**

A quasi-experimental design was adopted in this study. One hundred and twenty-three adults aged 60 or above were recruited from community centers through convenience sampling. They were allocated into two groups based on their level of depressive symptomatology as measured by the Geriatric Depression Scale (GDS). The intervention was to produce a written life story with pictures and memorabilia in four to six semi-structured sessions facilitated by trained volunteers. The outcome measures included general mental well-being (General Health Questionnaire, GHQ), life satisfaction (Life Satisfaction Scale Index A, LSI-A), and self-esteem (Rosenberg’s Self-esteem Scale, RSES). Data were collected at baseline (T0), immediately post-intervention (T1), and at the 3-month follow-up (T2). Generalized estimating equations were used to examine the effect of the intervention on the outcomes.

**Results:**

There was a significant interaction effect between the two groups at T1 (*β* = 0.244, *p* < 0.05) with improvements in the GHQ observed in the group with depressive symptomology. No significant time and interaction effects were seen on the LSI-A and RSES. The Friedman test was also used to examine whether the intervention itself would have any effects on the GDS score, with two groups combined. A reduction in the mean GDS score was found to be close to reaching a level of significance (χ^2^ = 5.912, *p* = 0.052).

**Conclusion:**

The findings of this study provided some preliminary evidence that life story work was effective at improving the general mental well-being of community-dwelling older adults with depressive symptomology. Because older adults with different levels of depressive symptoms might respond differently to life story work interventions, our findings offer interesting directions for future studies – for instance, on what population would benefit the most from Life Story Work and what would be the mechanism that renders Life Story Work effective.

## Background

Biographical, narrative, and reminiscence-based approaches to health and social care work with older adults have been very popular in the past several decades in various parts of the world. The biographical approach values the attitudes, interests, and desires of older adults as the culmination of a lifetime of experiences [1]. Some researchers, principally in the US [2] but also in the UK [3, 4], have demonstrated positive outcomes when older adults are provided with opportunities to recall and review their lives. More recently, many studies have shown that the biographical approach has positive impacts on the cognitive function or psychological well-being of older adults [5–7].

In this study, we aimed to examine effects of the use of life story books, a biographical approach, on improving three psychological indicators of older adults in the community, namely, self-esteem, life satisfaction, and general mental well-being. We also wanted to determine the differences in effect between those with and without depressive symptomatology.

### Biographical approaches

The biographical, narrative, and reminiscence-based approaches include specific reminiscences, life story work, and life review. A common element in the various biographical approaches is the possession of a life story unique to each individual [8]. Because reminiscence refers to a variety of different approaches, Professor Faith Gibson [4], an authority on reminiscence and life story work, urged researchers and clinicians to distinguish between general and specific reminiscence in order to better delineate the effects of their use in the field of gerontology. According to Gibson, general reminiscence is an approach that uses a variety of multi-sensory triggers to stimulate shared conversations on an agreed-upon topic or theme relating loosely to the known background and interests of the participants. Specific reminiscence, on the other hand, refers to the carefully selected, highly focused use of triggers known to closely approximate the detailed life history of the participant, and consistent efforts to stimulate recall during conversations.

In fact, life stories, in contrast to simple reminiscences, uncover much more than people’s past lives. They allow for an exploration of an individual’s more recent past, present, and plans and future concerns [1]. Coleman [8] cautioned us to distinguish between reminiscence and life review. A life review is the more intensive review of one’s life, which, more often than not, focuses on the emotionally charged problems in one’s life. Life reviews, therefore, should be facilitated by a professional [8].

Over time, more and more work has been done on using a person’s entire life story instead of a moment of remembering [9]. Atkinson [10] suggested that sharing one’s story is a way of purging, or releasing tension and burdens through validating personal experiences with others, which is central to the recovery process. It helps an individual to come to terms with his or her life [11]. The value of one’s life is affirmed when the feeling of connectedness with oneself and others occurs through the process of producing a life story record of some kind [12].

The application of biographical approaches as reported in the literature can be broadly classified to three population groups: that of community-dwelling seniors with symptoms of depression or those who might be at risk of suffering from depression [13], those with dementia who reside in nursing homes [14], and those with mental health problems and/or physical illnesses [15]. This paper focuses on the first group – life story work for the psychosocial well-being of community-dwelling seniors.

### Biographical approaches and psychological well-being

There have been many studies on the effects of life story work on those suffering from depression who live in institutions and in the community. For example, using a randomized controlled design Mastel-Smith and his colleagues [16] examined the effects that a community-based workshop on writing life stories had on 33 older adults suffering from depression. They reported that LSW was an effective intervention. The biographical approach was also found to be effective at improving other dimensions of psychological well-being, such as the self-esteem and life satisfaction of older adults in institutions [17, 18] and in the community [19].

In a randomized controlled trial (RCT), Chiang and his colleagues [18] evaluated whether an 8-week life review group program could improve the self-esteem and life satisfaction of men aged 75 and over living in a nursing home for veterans in Taiwan. Building on this study, the authors also reported the effects of specific reminiscence therapy on the depression, general psychological well-being, and loneliness of males aged 65 and over living in a nursing home [17]. The results of these studies showed that the biographical approach could be effective at improving the psychological well-being of older adults in institutions.

The effect of the biographical approach on psychological well-being was also seen in community samples, despite inconclusive evidence about its effectiveness. Lamers conducted an RCT to examine the effect of life reviews as an online-guided self-help intervention for adults aged 40 years and over with moderate symptoms of depression [19]. The results showed that the intervention was effective at improving the depressive symptoms, and the emotional and psychological well-being of both middle–aged and older adults. A study of life reviews employed among non-depressed older adults also reported a significant interaction effect on the depressive symptoms exhibited by community-dwelling older adults [20]. However, Fagerstrom [21] studied the use of individual or group-facilitated autobiography on older adults in retirement communities but observed no significant changes in both groups post-intervention.

Gibson highlighted that depressive individuals usually experience a loss of autonomy, confidence and control in their life. To them, reminiscence is a good mean of restoring control by controlling the process of recalling and reconstructing his/her own story [22]. Despite there is some evidence which showed a stronger beneficial impact of biographical approach on older adults with depression, there is currently lack of studies which directly compares the effect of LSB intervention on depressed and non-depressed older adults.

Based on the literature, we postulated that LSB would work more effectively on older adults with depressive symptomology. If the biographical approach is a useful intervention worthy of promotion on depressed older adults, professionals in the field need more and better evidence to inform them about its impact in practice. While in many studies the focus was on symptoms of depression as a major outcome of the biographical approach, we consider it is also important to examine the effect of life story work on other psychological variables, such as life satisfaction, self-esteem and general mental well-being.

The following questions were addressed in this study:

1. Would the introduction of the LSB intervention for community-dwelling seniors lead to an increase in their life satisfaction, self-esteem, and mental well-being?
2. Would the effect of the intervention, if any, be sustained 3-months post-intervention?
3. Which group of older adults (with or without depressive symptoms) would benefit more from the intervention?

## Methods

### Design

This study examined whether the production of a life story book (LSB) – a record of a person’s life history – would lead to a higher level of life satisfaction and self-esteem in older adults in the community. This was a quasi-experimental study that compared the outcomes between two groups (one with depressive symptoms and one without) at baseline (T_0_), immediately post-intervention (T_1_), and 3 months post-intervention (T_2_).

### Participants

Convenience sampling was employed and the study settings were community centers for seniors in the community. The participants were community-dwelling older adults aged 60 and over (in Hong Kong, anyone aged 60 or over can join a center for older adults in their neighborhood), who were able to communicate most of the time (according to the criteria of the Resident Assessment Instrument [RAI] communication scale [23]; and able to understand and speak Cantonese (the dominant Chinese dialect used in the southern Chinese province of Guangdong and Hong Kong). Excluded were those with active major psychiatric illnesses, including schizophrenia, bipolar disorders, and depression; those with any acute or unstable chronic medical conditions including cardiac or lung diseases; those with any active psychosocial crises such as bereavement or relationship problems; those who were blind; and those who were unable to hear even with hearing aids [23]. Eligible seniors were recruited and allocated into two groups. Group 1(G1) consisted of older adults who had a Geriatric Depression Scale (GDS) score of less than 5 (i.e., no symptoms of depression); the second (G2) consisted of older adults with minor depressive symptoms (GDS scores of 5 and 7, with GDS 8 or above being the cut-off score for identifying depressive symptomatology that warrants medical attention [24].

In previous studies [5, 25], the reminiscence approach was shown to have an effect size of 0.5 in improving well-being on older adults. With a significance level (alpha) of 0.05, an effect size of 0.5 and correlation of repeated measure to be 0.4, a sample of 38 could achieve 80% power to detect between group difference and changes at three time-points (pre-intervention, post intervention and follow up). Previous studies on older individuals using the LSB approach revealed a 10 to 15% attrition rate. The final sample size was determined to be 48 participants for each group.

### Intervention

The life story approach for the writing of life story books developed by Lai [26] was used. The LSB approach incorporates a person’s development over the course of his/her lifetime (i.e., from childhood to older age), and his/her psychosocial development and ways of life (e.g., education, marriage, career choices, and hobbies). The production of the LSB is a planned intervention to facilitate the telling or sharing of past events in a person’s life. It eliminates the requirements of a rigid structure such as that of a biography and allows the person to tell his or her story in a more relaxed manner, similar to engaging in a dialogue with an acquaintance.

A trained student assistant helped each senior to compile the senior’s life story if he or she were illiterate. The student assistants would scan the seniors’ photographs, take pictures of objects with special meaning as inserts into the LSB, or find pictures related to the stories being told to make the LSB livelier and more colorful (e.g., pictures of the person’s hometown). Four to six meetings were needed for the seniors and the student assistants to discuss and produce the LSB [26]. These meetings were conducted mostly in the community centers and sometimes in the seniors’ home when the seniors could not go to the centers because of their physical status. The duration of these meetings was scheduled as 60 min. The end product of the intervention was a written story with pictures or other memorabilia. The total number of words, pages and pictures of completed life story books varied among the participants. The same approach was adopted when facilitating the participants to produce their life story books. Still, the end product were influenced by how the individual would like him/herself to be represented in his or her own life story book. The total number of meetings varied among the participants because of the same reason.

The volunteers were university students from the nursing department who had completed at least 80% of the required training, which was comprised of a 2-hour hands-on session held once a week for 6 weeks. Throughout the training program, the student assistants met with the trainer for guidance and feedback.

### Instrumentation

The basic demographic and clinical characteristics of the participants were collected to facilitate the detection of any significant differences within and between groups. These included data on their gender, age, date of birth, education, and number of medical diagnoses. The functional abilities of the participants were assessed using Lawton’s Instrumental Activities of Daily Living scale [27]. The GDS short form was used to detect symptoms of depression [28].

Life satisfaction and a person’s perception of him/herself can be affected by his/her life circumstances. To understand whether any outside impact would likely affect the person’s life, the Revised Life Events Scale [29] were administered to the participants. In addition, the participants were asked if they were experiencing any financial strain [30].

The outcome measures included the Life Satisfaction Scale Index A (LSI-A) [31]; the Rosenberg’s Self-esteem Scale (RSES) [18]; and the General Health Questionnaire-30 (GHQ) [32]. The GHQ is one of the most commonly used assessments of mental well-being [33].

All of the aforementioned measures had good psychometric properties and had been validated for use in the Hong Kong Chinese population.

Ethical approval to conduct the study was obtained from the Human Subjects Ethics Sub-committee of the School of Nursing, The Hong Kong Polytechnic University (equivalent to the Institutional Review Boards in Western countries). Because some of the participants in G2 were experiencing some minor symptoms of depression, the team instituted a protocol to support them in order to prevent any untoward events or outcomes. Research personnel, including the student assistants, were trained to become sensitive to the participants’ cues, for example, exhibiting behaviors that indicated that they did not want to continue or were unsuitable to continue with the session or project. A back-up and referral system was in place for the participants should they become emotionally disturbed or depressed during the course of the study.

### Procedures

Eligible participants of the social centers were approached and their written informed consent was obtained. Recruited participants were assigned to either G1 or G2 based on the inclusion and exclusion criteria. A research assistant who was not involved in the delivery of the intervention collected the assessment data. During each visit the volunteer would establish a quiet and relaxed atmosphere at the social center, ensuring that the G1 or G2 participant felt comfortable and could engage in the processes without interruptions. The student assistants would facilitate the process with the use of semi-structured questions about the life of the participant, which was developed by the first author of the team. The student visited each participant weekly for 4 to 6 weeks or until the LSB was completed.

### Data analysis

SPSS 24 for Windows was used to manage and analyze the data. Descriptive statistics were used to summarize the characteristics of the sample. The sample characteristics at baseline for those who did not have depressive symptoms versus those who had were compared. Nonparametric tests were adopted because of the relatively small sample size. The Mann-Whitney U test was used to examine the difference between groups for continuous variables, whereas the Chi-square test and Fisher’s Exact test were used for categorical variables. For the multiple response variables, the Chi-square statistics were adjusted with the first-order Rao-Scott correction. To minimize the potential risk of confounding and selection bias among the variables, ANCOVA (Analysis of Covariance) was used on baseline data to select the variables to be included in the multivariate Generalized Estimating Equation (GEE) model for further analysis. The variables that were found to be significant at a *p* value of < 0.05 in ANCOVA were selected as co-variates. GEE modeling was used to measure changes in outcomes at each time point in relation to the baseline. All models were adjusted for covariates, which included age, sex, income, religion, and physical functioning level (BI). All outcomes were analyzed with a first-order auto regression covariance structure and found to have fitness. GEE is one of the most efficient methods to use with small unbalanced longitudinal data [34]. For the treatment of non-response items and data related to loss to follow up, the last observation carried forward approach was adopted. Missing data of LSI-A, RSES and GHQ were 3.5%. Education level, religion and income were found to have missing data, ranging from 1.8 to 3.5%. These missing data were replaced by modal value. Because the groups were classified according to the participants’ GDS score, GDS scores could therefore not be included in the GEE modeling. To examine if there were changes in the GDS score over time, the Friedman test was used. A two-sided level of significance, *p* < 0.05, was applied for all tests.

## Results

**Fig. 1:**
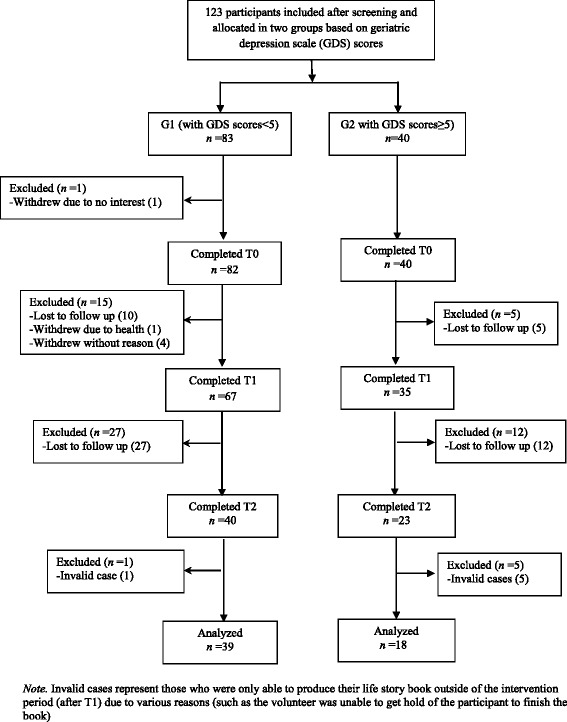
Flow chart of the study. Flow chart diagram showing participants flow through each stage of the quasi-experimental study, including enrollment, intervention allocation, follow-up (T0, T1, T2) and data analysis

A total of 123 participants were recruited at baseline but only 57 participants completed both the intervention and follow-up assessment at T2 (Fig. 1). Many participants (81%) were lost to follow-up either because they refused to continue or could not be contacted after repeated attempts (e.g., not returning calls). Other reasons include withdrawal due to health problems or no interest. The baseline and outcome variables between those who completed the study and those who dropped out were compared (Table 1). No statistical differences were found except that a greater proportion of subjects withdrew (86.4%) were satisfied with general support received when compared with those completed the intervention (66.7%) at T0, χ^2^ = 6.348, *df* = 1, *p* = 0.012.

**Table 1:** Dropout analysis

		Withdraw (*n* = 60)	Case (*n* = 57)	χ^2^, df, *P* value
		n / %	n / %	
Age (mean, SD, median, range)		75.34, 8.057, 75.00, 60–95	74.56, 8.089, 74.00, 60–90	U = 1550, *p* = 0.467^+^
Gender	Male	15 / 25.4	8 / 14.0	χ^2^ = 2.365, df = 1,
	Female	44 / 74.6	49 / 86.0	*p* = 0.124
Education level	No education	17 / 29.8	20 / 36.4	χ^2^ = 0.995, df = 2,
	Primary	27 / 47.4	21 / 38.2	*p* = 0.608
	Secondary & tertiary	13 / 22.8	14 / 25.5	
Religion	None	25 / 52.1	20 / 37.0	χ^2^ = 2.510, df = 2,
	Christianity & Catholicism	8 / 16.7	10 / 18.5	*p* = 0.285
	Oriental religions	15 / 31.3	24 / 44.4	
Marital status	Not married	37 / 63.8	32 / 56.1	χ^2^ = 0.702, df = 1,
	Married	21 / 36.2	25 / 43.9	*p* = 0.402
Employment status	Retired	34 / 57.6	25 / 43.9	χ^2^ = 4.124, df = 3,
	Blue collar	12 / 20.3	14 / 24.6	*p* = 0.248
	White collar	9 / 15.3	8 / 14.0	
	Others	4 / 6.8	10 / 17.5	
Income	Without	22 / 39.3	26 / 46.4	χ^2^ = 0.583, df = 1,
	With	34 / 60.7	30 / 53.6	*p* = 0.445
ADL difficulty	No difficulty	50/ 87.7	45 / 78.9	χ^2^ = 1.579, df = 1,
				*p* = 0.209
Frequency of meeting with close relatives	< 1 / month	19 / 32.8	19 / 33.3	χ^2^ = 10.094,
	1 / month	10 / 17.2	16 / 28.1	df = 5, *p* = 0.073
	> 1 / month	10 / 17.2	7 / 12.3	
	1 / week	2 / 3.4	8 / 14.0	
	> 1 / week	7 / 12.1	4 / 5.5	
	Daily	10 / 17.2	3 / 5.3	
Frequency of meeting with close friends	< 1 / month	12 / 20.3	12 / 21.1	χ^2^ = 8.110, df = 5,
	1 / month	5 / 8.5	6 / 10.5	*p* = 0.150
	> 1 / month	10 / 16.9	2 / 3.5	
	1 / week	10 / 16.9	6 / 10.5	
	> 1 / week	10 / 16.9	16 / 28.1	
	Daily	12 / 20.3	15 / 26.3	
Satisfaction about general support	Unsatisfied/Neutral	8 / 13.6	19 / 33.3	χ^2^ = 6.348, df = 1,
	Satisfied	51 / 86.4	38 / 66.7	p = 0.012
BI at T0 (mean, SD, median, range)		19.63, 1.272, 20.00, 11–20	19.74, 0.835, 20.00, 15–20	U = 1630.000, *p* = 0.651^+^
GDS at T0 (mean, SD, median, range)		3.00, 2.181, 2.00, 0–7	3.23, 2.236, 3.00, 0–7	U = 1588.500, *p* = 0.603^+^
Life Events at T0 (mean, SD, median, range)		1.00, 1.000, 1.00, 0–4	1.02, 0.876, 1.00, 0–4	U = 1610.000, *p* = 0.671^+^
Life Satisfaction at T0 (mean, SD, median, range)		11.83, 3.425, 13.00, 3–16	12.05, 3.125, 13.00, 3–17	U = 1656.500, *p* = 0889^+^
Self Esteem at T0 (mean, SD, median, range)		7.20, 2.538, 8.00, 0–10	7.25, 2.029, 8.00, 1–10	U = 1590.500, *p* = 0.610^+^
GHQ at T0 (mean, SD, median, range)		23.03, 6.948, 21.00, 12–48	22.28, 7.632, 22.00, 9–45	U = 1519.000, *p* = 0.368^+^

Note. The above table is based on the number of valid cases (*N* = 117) we recruited at the beginning of the study.; +, Mann-Whitney U test; BI-Barthel Index; GDS-Geriatric Depression Scale, GHQ – General Health Questionnaire-30

Among those who completed the intervention, 39 were without depressive symptoms (G1) while 18 were experiencing depressive symptoms (G2). Their mean age was 74.56 ± 8.09, and 49 (86%) of them were female. Thirty-two of them (56.1%) were single (widowed, separated, or never married). The majority (68.4%) lived with their relatives. Most had either a primary level of education (*n* = 21, 38.2%) or no education (*n* = 20, 36.4%). About 60% of them were either a believer of an oriental religion (such as ancestral worship or Buddhism) or believed in Christianity (*n* = 34, 62.9%). Over 40 % reported that they had no formal occupation or had not worked before (*n* = 25, 43.9%), 14 (24.6%) were in blue collar occupations, and almost a third were in white collar or other (such as business) occupations (*n* = 18, 31.5%). While 26 of them (46.4%) reported having no income, the remainder had a regular income from either their children, savings, a pension, or social security (e.g., old age allowance). Even though nearly half of them did not have a regular income, few were worried about their financial situation (*n* = 6, 10.5%). Thirty-seven of them (64.9%) had no sleep problems. Most experienced no difficulties with IADL (*n* = 45, 78.9%). The majority exercised (*n* = 50, 87.7%), watched television (*n* = 50, 87.7%), participated in volunteer services (*n* = 39, 68.4%) or traveled (*n* = 34, 59.6%) in their free time.

At baseline, there were no significant differences between the groups with the exception for just three variables, namely whether they had sleeping problems (χ^2^ = 7.900, *p* = 0.014), their physical functioning level (G1: 19.92 vs. G2: 19.33, *z* = 6.08, *p* < 0.001), and their satisfaction with the overall support that they received from the people around them (χ^2^ = 9.546, *p* = 0.009). Table 2 is a summary of the sample profile.

**Table 2:** Sample characteristics at baseline

	Overall	G1 (*n* = 39)	G2 (*n* = 18)	*p*
	(*n* = 57)			
	*n* (%)	*n* (%)	*n* (%)	
Age (*M*, *SD*)	74.56, 8.09	74.36, 7.70	75.00, 9.10	0.829^a^
Gender				
Male	8 (14.0)	7 (17.9)	1 (5.6)	0.414^c^
Female	49 (86.0)	32 (82.1)	17 (94.4)	
Marital status				
Single	32 (56.1)	21 (53.8)	11 (61.1)	0.607^b^
Married	25 (43.9)	18 (46.2)	7 (38.9)	
Dwelling status				
Live alone	18 (31.6)	13 (33.3)	5 (27.8)	0.675^b^
Live with relatives	39 (68.4)	26 (66.7)	13 (72.2)	
Education level				
No education	20 (36.4)	10 (26.3)	10 (58.8)	0.068^c^
Primary	21 (38.2)	17 (44.7)	4 (23.5)	
Secondary or above	14 (25.5)	11 (28.9)	3 (17.6)	
Religion				
None	20 (37.0)	11 (30.6)	9 (50.0)	0.159^c^
Christianity & Catholicism	10 (18.5)	9 (25.0)	1 (5.6)	
Oriental religions	24 (44.4)	16 (44.4)	8 (44.4)	
Employment status				
Not work before	25 (43.9)	13 (33.3)	12 (66.7)	0.110^c^
Blue collar	14 (24.6)	12 (30.8)	2 (11.1)	
White collar	8 (14.0)	7 (17.9)	1 (5.6)	
Others	10 (17.5)	7 (17.9)	3 (16.7)	
Income				
Without	26 (46.4)	15 (39.5)	11 (61.1)	0.129^b^
With	30 (53.6)	23 (60.5)	7 (38.9)	
Fear for financial difficulty				
No	51 (89.5)	37 (94.9)	14 (77.8)	0.072^c^
Yes	6 (10.5)	2 (5.1)	4 (22.2)	
IADL with difficulty				
No	45 (78.9)	33 (84.6)	12 (66.7)	0.165^c^
Yes	12(21.1)	6 (15.4)	6 (33.3)	
Number of medical diagnosis (*M, SD*)	1.44, 1.337	0.92, 0.929	2.56, 1.439	0.005^a^
Leisure activities+				
Exercise (Yes/No)	50 (87.7)	35 (89.7)	15 (83.3)	0.241^d^
Travel	34 (59.6)	25 (64.1)	9 (50.0)	
Voluntary work	39 (68.4)	31 (79.5)	8 (44.4)	
Watch TV	50 (87.7)	35 (89.7)	15 (83.3)	
Sleeping problem				
None	37 (64.9)	30 (76.9)	7 (38.9)	0.014^c*^
1–4/week	15 (26.3)	7 (17.9)	8 (44.4)	
> 4/week	5 (8.8)	2 (5.1)	3 (16.7)	
Satisfaction about general support				
Unsatisfied	3 (5.3)	0 (.0)	3 (16.7)	0.009^c*^
Neutral	16 (28.1)	9 (23.1)	7 (38.9)	
Satisfied	38 (66.7)	30 (76.9)	8 (44.4)	
GDS (*M, SD*)	3.23, 2.236	1.95, 1.372	6.00, 0.840	0.000^a**^
BI (*M, SD*)	19.74, 0.835	19.92, 0.27	19.33, 1.372	0.033^a*^
LIS-A (*M, SD*)	12.10, 3.132	12.92, 2.667	10.31, 3.385	0.005^a*^
RSES (*M, SD*)	7.25, 2.029	7.97, 1.460	5.67, 2.223	0.000^a**^
GHQ (*M, SD*)	22.31, 7.629	20.32, 6.298	26.61, 8.624	0.005^a*^

G1: Participants without depressive symptoms; G2: Participants with depressive symptoms

*IADL* instrumental activities of daily living, *GDS* geriatric depression scale, *LIS-A* life satisfaction scale, *RSES* Rosenberg’s Self-Esteem Scale, *GHQ* general health questionnaire-30, *BI* Barthel Index

^+^Allow to choose more than one option

^*^p < 0.05;^**^p < 0.005

^a^Mann Whitney U Test

^b^Chi-Square Test

^c^Fisher Exact Test

^d^First-order Rao-Scott Corrected Chi-square

Group comparisons for outcome variables were also conducted at baseline. Participants in G1 had significantly lower scores in the GHQ (G1: 20.32 vs. G2: 26.61, z = 2.833, *p* = 0.005), and for life satisfaction (G1: 12.92 vs. G2: 10.31, *z* = 2.808, *p* = 0.005) and self-esteem (G1: 7.79 vs. G2: 5.67, z = 3.654, *p* < 0.001) (Table 2).

The changes in the outcome variables by groups over time are shown in Table 3. In the multivariate GEE analyses, a significant interaction effect (time x group) between the two groups was found only in the GHQ score at T1. Significant between-group improvements in the RSES score were seen for both groups (G1: 7.79 at T0 and 8.97 at T2; G2: 5.67 at T0 and 6.22 at T2), but no significant difference was observed in the time and group interaction effect. Also, no significant interaction effect in the LSI-A was found (Table 4).

**Table 3:** Outcome Measures by Groups over Time

Measure/Group	T0			T1		T2	
	*n*	*M / SD*	Median [Range]	*M / SD*	Median [Range]	*M / SD*	Median [Range]
Life Satisfaction Scale							
G1	39	12.92 / 2.667	13.00 [5–17]	13.32 / 2.420	14.00 [8–17]	14.15 / 3.432	15.00 [2–18]
G2	18	10.31 / 3.385	10.00 [3–15]	9.78 / 3.671	10.00 [3–15]	10.28 / 4.800	12.00 [2–16]
Rosenberg’s Self-Esteem Scale							
G1	39	7.79 / 1.460	8.00 [5–10]	8.04 / 1.680	9.00 [4–10]	8.97 / 1.308	9.00 [4–10]
G2	18	5.67 / 2.223	5.50 [1–9]	5.83 / 3.073	6.50 [0–10]	6.22 / 3.209	7.50 [0–9]
General Health Questionnaire-30							
G1	39	20.32 / 6.298	18.00 [9–39]	22.34 / 6.389	22.00 [11–40]	19.08 / 4.492	19.00 [9–35]
G2	18	26.61 / 8.624	24.50 [12–45]	23.17 / 6.447	23.50 [13–35]	26.22 / 6.744	26.00 [14–38]

G1: Participants without depressive symptom; G2: Participants with depressive symptom

*SD* standard deviation

**Table 4:** GEE^#^ Analysis – Changes in Outcome Measures^+^ at Post-intervention Compared Against Baseline between Groups

	LIS-A			RSES			GHQ		
	Beta	*SE*	95% CI	Beta	*SE*	95% CI	Beta	*SE*	95% CI
Time sequence									
T2 vs T0	−0.051	0.116	[−0.279, 0.178]	0.050	0.112	[− 0.168, 0.269]	0.012	0.090	[− 0.164, 0.189]
T1 vs T0	−0.018	0.100	[−0.213, 0.177]	0.031	0.111	[−0.187, 0.250]	−0.147	0.090	[−0.324, 0.031]
Group									
G1 vs G2	0.136	0.076	[−0.012,0.285]	0.252^**^	0.075	[0.1044, 0.399]	−0.200^*^	0.088	[−0.373, − 0.028]
Group in time sequence (G1 vs G2)									
T2 vs T0	0.140	0.127	[−0.109,0.389]	0.053	0.118	[−0.177, 0.284]	−0.072	0.107	[−0.282, 0.137]
T1 vs T0	0.043	0.109	[−0.170, 0.256]	− 0.030	0.119	[− 0.263, 0.204]	0.244^*^	0.110	[0.028, 0.461]
Gender (Female = 1, Male = 0)	−0.095^*^	0.042	[−0.177,-0.013]	− 0.007	0.045	[− 0.095, 0.081]	0.079	0.055	[−0.028, 0.186]
Income (Yes = 1, No = 0)	−0.022	0.044	[−0.109,0.064]	− 0.065	0.040	[− 0.143, 0.014]	−0.042	0.048	[−0.136, 0.053]
Religion (Yes = 1, No = 0)	0.101^*^	0.044	[0.015,0.186]	0.076	0.042	[−0.007, 0.158]	−0.029	0.053	[−0.133, 0.076]
Age	−0.003	0.003	[0.009, 0.002]	−0.007^*^	0.003	[−0.012, − 0.001]	0.005^*^	0.003	[0.0002, 0.011]
BI	0.128^**^	0.034	[0.061, 0.195]	0.097^**^	0.031	[0.0359, 0.158]	−0.057^*^	0.025	[−0.107, − 0.007]

# GEE used to test difference between groups on mean change between baseline and follow up adjusting for age, gender, income, religion and Barthel Index

+ Logarithm transformation was taken for outcome variables in the analysis

G1: Participants without depressive symptoms; G2: Participants with depressive symptoms

*SE* standard error, *LIS-A* life satisfaction scale, *RSES* Rosenberg’s Self-Esteem Scale, *GHQ* general health questionnaire-30, *BI* Barthel Index

^*^p < 0.05; ^**^ *p* < 0.00

GDS scores were collected at all time points as a control variable. We also tested to determine whether there were changes in the GDS score over time. The difference in the mean scores almost reached statistical significance at T2 (χ^2^ = 5.912, *p* = 0.052) (Table 5).

**Table 5:** Geriatric Depression Scale over Time

		T0	T1	T2	*p*
Geriatric Depression Scale	N	57	57	57	0.052
	Mean	3.23	3.91	2.96	
	*SD*	2.236	3.302	3.117	
	Median	3.00	3.00	2.00	
	Range	[0–7]	[0–14]	[0–13]	

*SD* standard deviation

We examined whether completing the life story book by oneself (*n* = 15) or with the help of volunteers (*n* = 42) would have any effect on the outcomes. The same GEE model testing was performed, with those who self-completed their life story book assessed as one group and those who completed their LSB with the help of volunteers assessed as another group. The same finding on the GHQ, i.e., a significant interaction effect (time x group) between-group difference, was observed at T1. The improvement in the GHQ was found in those who completed their LSB with the help of volunteers. In this analysis, an interaction effect of RSES was observed at T2. A greater improvement in the mean RSES score was observed from those who had self-completed their LSB (Self–completed Group: T2-T0, change score 1.47; Volunteer-facilitated Group: T2-T0, change score 0.64).

## Discussion

### Effect on general mental well-being

Our result showed that LSBs are effective at improving the general mental well-being of G2 when compared with G1, as reflected by the significant interaction effect shown on GHQ scores. A similar finding was also found in the study [19], which demonstrated that an online self-help life review intervention is effective at improving mental well-being scores as measured by the Mental Health Continuum - Short Form. However, their samples included middle-aged adults, and they targeted adults with more severe symptoms of depression, making direct comparisons to our study inappropriate. There is another study using life story work, which reported an improvement in general well-being, but the sample was comprised of people with dementia in nursing homes [25].

Our LSB intervention employed a structured life story production process that allowed older adults to review various stages in their lives from childhood to late adulthood. According to Wills and Days [35], this process enabled older adults to reflect on their past experiences and gave them an opportunity to positively affirm themselves and their sense of self, thereby enhancing their overall well-being. According to Besser and Priel [36], depressed older adults were often beset with questions related to self-identity. The G2 of our study had a slightly higher level of depressive symptomatology. We postulated that the processes of LSB might have provided insights and positive feedback about the participants’ self-concept, which led to the observed result.

### Effects on self-esteem and life satisfaction

Our study showed there was no improvement in self-esteem in the sample. A similar finding was found in some interventional studies that targeted community-dwelling older adults [37, 38]. Chung [37] assessed the effectiveness of reminiscence therapy with the use of life story book on community-dwelling older adults and found no improvement in self-esteem after the intervention. A large-scale randomized controlled trial also reported that reminiscence therapy did not improve the self-esteem of community-dwelling older adults with depressive symptomology [38]. Some studies, however, came to a different conclusion. In the study conducted by Chiang and his colleagues [18], a life review group was found to be effective at enhancing the self-esteem of older adults in a veterans’ home, while a meta-analysis [6] on reminiscence interventions and life reviews showed a small effect on self-esteem (*d* = 0.20) among the 22 studies that they reviewed. Our secondary analysis on the question of whether a volunteer-facilitated production of an LSB would make any difference revealed that those participants who were able to complete their LSB by themselves had a more favorable response to the intervention in terms of their self-esteem.

Our results also showed there was no significant improvement in life satisfaction. We are not the only study to have found insignificant results on life satisfaction from using a life story approach. Latorre’s study [20] targeting community-dwelling older adults found no significant interaction effect on life satisfaction when they compared a life review group with an education group. Chin [39] carried out a meta-analysis of four studies on reminiscence therapy targeting older adults. His results indicated that that reminiscence therapy did not have any beneficial effects on life satisfaction. Yet, a different conclusion was reached from two systematic reviews of both reminiscence interventions and life reviews. Bohlmeijer, Smit, and Cuijper [40] reviewed 17 studies and found a moderate effect size of 0.54 on life satisfaction, whereas a more recent review [6] also found a small effect on life satisfaction (*d* = 0.22).

In summary, the current evidence of life story work on self-esteem and life satisfaction is inconclusive. The literature suggested that self-esteem is a trait that is relatively stable and enduring over time [41] and life satisfaction is a broadly defined construct [42]. The clinical effect on these constructs is therefore less likely to be detectable [43, 44]. Pavot and Diener [42] suggested that to detect changes in life satisfaction, the effect of the intervention has to be profound. This could be a possible explanation for why the current evidence on these two constructs is inconclusive. In this study, our sample size was small and likely with inadequate power. The effect of the life story work intervention might also not yet have reached a high enough dose to produce a therapeutic effect.

We also examined if life story work would lead to any changes in the level of the GDS score in our study. The reduction in the GDS was found to be close to reaching a level of significance when the two groups (G1 and G2) were combined to investigate if there were changes in the GDS score over time. Our result is consistent with the result of intervention studies targeting community-dwelling older adults [37, 38, 44]. For example, Chan and her colleagues [44] conducted a life story review with a life story book and found a reduction in symptoms of depression in community-dwelling older adults post-intervention. It is reasonable to expect this improvement in the GDS since to older adults life story work is an enjoyable process and a meaningful activity [45, 46]. This enjoyment relates particularly to the sharing of their story and to being heard, having meaningful communication throughout the process [44], and reminding older adults of the good things that they had forgotten [46].

This study has several limitations that must be taken into consideration. First, the present study was a quasi-experimental study using convenience sampling, which limits the external validity of the study. The observed results need to be further investigated using more rigorously designed studies, such as RCT. Second, the small size of the sample also limited the statistical power of the study. Caution is needed when interpreting the results. Third, our entire sample consisted of community-dwelling older adults who were relatively healthy; this again limits the generalizability of our findings. Future studies with a larger sample size and further examination of whether satisfaction with general support received by older adults would make a difference will be needed. Older adults who are vulnerable such as those with chronic illnesses or clinically depressed also need to be included in randomized controlled studies.

## Conclusions

To conclude, this study provided evidence of the effect of LSBs on the general mental well-being of community-dwelling older adults with depressive symptomatology. LSBs could also have an impact on reducing symptoms of depression in community-dwelling older adults. This study provides some directions for a future study investigating the mechanism behind LSBs.

## Abbreviations

G1: Older adults who had a GDS score of less than 5
G2: Older adults who had a GDS score of 5 or above
GDS: Geriatric depression scale
GHQ: General health questionnaire
LSB: Life story book
LSI-A: Life satisfaction scale index A
RSES: Rosenberg’s self-esteem scale

## References

[1] Clarke A (2000). Using biography to enhance the nursing care of older people. Br J Nurs.

[2] Haight BK, Webster JD (1995). Art and science of reminiscing: theory, research, methods and applications.

[3] Coleman PG (1986). Aging and reminiscence processes: social and clinical implications.

[4] Gibson F, Bornat J (1994). What can reminiscence contribute to people with dementia. Reminiscence reviewed: evaluations, achievements, perspectives.

[5] Bohlmeijer E, Roemer M, Cuijpers P, Smit F (2007). The effects of reminiscence on psychological well-being in older adults: a meta-analysis. Aging Ment Health.

[6] Pinquart M, Forstmeier S (2012). Effects of reminiscence interventions on psychosocial outcomes: a meta-analysis. Aging Ment Health.

[7] Syed Elias SM, Neville C, Scott T. The effectiveness of group reminiscence therapy for loneliness, anxiety and depression in older adults in long-term care: a systematic review. Geriatr Nurs. 2015; 10.1016/j.gerinurse.2015.05.004. 10.1016/j.gerinurse.2015.05.004 26099638

[8] Coleman P, Bornat J (1994). Reminiscence within the study of ageing: the social significance of story. Reminiscence reviewed: evaluations, achievements, perspectives.

[9] Hendrix S, Haight BK, Webster JD, Haight BK (2002). A continued review of reminiscence. Critical advances in reminiscence work: from theory to application.

[10] Atkinson R (1998). The life story interview.

[11] Plastow NA (2006). Libraries of life: using life history books with depressed care home residents. Geriatr Nurs.

[12] Hellen CR (1998). Alzheimer’s disease: activity-focused care.

[13] Symes L, Mastel-Smith B, Hersch G, Binder B, Malecha A, McFarlane J (2007). The feasibility of home care workers delivering an intervention to decrease depression among home-dwelling, older women: a qualitative analysis. Issues Ment Health Nurs.

[14] Grøndahl VA, Persenius M, Bååth C, Helgesen AK. The use of life stories and its influence on persons with dementia, their relatives and staff - a systematic mixed studies review. BMC Nurs. 2017. 10.1186/s12912-017-0223-5. 10.1186/s12912-017-0223-5 PMC5457564 28588424

[15] Korte J, Bohlmeijer ET, Smit F. Prevention of depression and anxiety in later life: design of a randomized controlled trial for the clinical and economic evaluation of a life-review intervention. BMC Public Health. 2009. 10.1186/1471-2458-9-250. 10.1186/1471-2458-9-250 PMC2721846 19619284

[16] Mastel-Smith B, McFarlane J, Sierpina M, Malecha A, Haile B (2007). Improvin g depressive symptoms in community-dwelling older adults: a psychosocial intervention using life review and writing. J Gerontol Nurs.

[17] Chiang KJ, Chu H, Chang HJ, Chung MH, Chen CH, Chiou HY, Chou KR. The effects of reminiscence therapy on psychological well-being, depression, and loneliness among the institutionalized aged. Int J Geriatr Psychiatry. 2010. 10.1002/gps.2350. 10.1002/gps.2350 19697299

[18] Chiang K, Lu R, Chu H, Chang Y, Chou K (2008). Evaluation of the effect of a life review group program on self-esteem and life satisfaction in the elderly. Int J Geriatr Psychiatry..

[19] Lamers SM, Bohlmeijer ET, Korte J, Westerhof GJ. The efficacy of life-review as online-guided self-help for adults: a randomized trial. J Gerontol B Psychol Sci Soc Sci. 2015. 10.1093/geronb/gbu030. 10.1093/geronb/gbu030 24691155

[20] Latorre JM, Serrano JP, Ricarte J, Bonete B, Ros L, Sitges E (2015). Life review based on remembering specific positive events in active aging. J Aging Health.

[21] Fagerstrom K (2012). The differential effects of guided autobiography types on well-being in the elderly.

[22] Gibson F (2011). Reminiscence and life story work: a practical guide.

[23] Chou KL, Chi I, Leung ACT, Wu YM, Liu CP (2001). Validation of minimum data set for nursing home in Hong Kong Chinese elders. Clin Gerontol.

[24] Woo J, Ho SC, Lau J, Yuen YK, Chiu H, Lee HC, Chi I (1994). The prevalence of depressive symptoms and predisposing factors in an elderly Chinese population. Acta Psychiatr Scand.

[25] Lai CKY, Chi I, Kayser-Jones J (2004). A randomized controlled trial of a specific reminiscence approach to promote the well-being of nursing home residents with dementia. Int Psychogeriatr.

[26] Lai CKY. Improving the quality of life for nursing home residents with dementia: a life story approach, Unpublished doctoral dissertation: The University of Hong Kong; 2003.

[27] Tong AYC, Man DWK (2002). The validation of the Hong Kong Chinese version of the Lawton instrumental activities of daily living scale for institutionalized elderly persons. OTJR.

[28] Chiu HFK, Lee HCB, Wing YK, Kwong PK, Leung CM, Chung DWS (1994). Reliability, validity and structure of the Chinese geriatric depression scale in a Hong Kong context: a preliminary report. Singap Med J.

[29] Boey KW, Chi I (1998). A study of life event and psychological well-being of the odler adults in Hong Kong. J Clin Geropsy.

[30] Chou KL, Chi I (1999). Determinants of life satisfaction in Hong Kong Chinese elderly : a longitudinal study. Aging Ment Health.

[31] Chi I, Boey KW (1992). Validation of measuring instruments of mental health status of the elderly in Hong Kong.

[32] Chan DW (1995). The two scaled versions of the Chinese general health questionnaire: a comparative analysis. Soc Psychiatry Psychiatr Epidemiol.

[33] Jackson C (2007). The general health questionnaire. Occup Med.

[34] Muth C, Bales KL, Hinde K, Maninger N, Mendoza SP, Ferrer E (2016). Alternative models for small samples in psychological research: applying linear mixed effects models and generalized estimating equations to repeated measures data. Educ Psychol Meas.

[35] Wills T, Day MR (2008). Valuing the person’s story: use of life story books in a continuing care setting. Clin Interv Aging.

[36] Besser A, Priel B (2005). Interpersonal relatedness and self-definition in late adulthood depression: personality predispositions, and protective factors. Soc Behav Pers.

[37] Chung CC (2008). An intergenerational reminiscence programme for older adults with early dementia and youth volunteers: values and challenges. Scand J Caring Sci.

[38] Zhou W, He G, Gao J, Yuan Q, Feng H, Zhang CK (2012). The effects of group reminiscence therapy on depression, self-esteem, and affect balance of Chinese community dwelling elderly. Arch Gerontol Geriatr.

[39] Chin MH (2007). Clinical effect of reminiscence therapy in older adults: a meta-analysis of controlled trials. HKJOT.

[40] Bohlmeijer E, Smit F, Cuijpers P (2003). Effects of reminiscence and life review on late-life. Int J Geriatr Psychiatry..

[41] Lyubomirsky S, Tkach C, Dimatteo MR (2006). What are the differences between happiness and self-esteem?. Soc Indic Res.

[42] Pavot W, Diener ED (2008). The satisfaction with life scale and the emerging construct of life satisfaction. J Posit Psychol.

[43] Blascovisch J, Tomaka J, Robinson JP, Shaver PR, Wrightsman LS, Andrews FM (1991). Measures of self-esteem. Measures of personality and social psychological attitudes.

[44] Chan MF, Leong SP, Heng BL, Mathew BK, Khan AL, Lourdusamy SS, Nagapan M, Woo SF, Chee WY, Ho CM, Taylor BJ (2014). Reducing depression among community dwelling older adults using life story review: a pilot study. Geriatr Nurs.

[45] McKeown J, Clarke A, Repper J (2006). Life story work in health and social care: systematic literature review. J Adv Nurs.

[46] Morgan S, Woods RT (2011). Life review with people with dementia in care homes: a preliminary randomized controlled trials. Nonpharmacol Ther Dement.

